# Comparison between sleep-deprived, and melatonin-induced sleep electroencephalography in children of different ages: a randomized controlled trial

**DOI:** 10.1016/j.cnp.2025.09.002

**Published:** 2025-09-18

**Authors:** Greta Gustafsson, Martin Ulander, Cornelia Lauermann, Johanna Thegerström, Kathe Dahlbom, Anders Broström, Eva Svanborg, Magnus Vrethem

**Affiliations:** a Region Östergötland, Clinical Neurophysiology, Neurobiology, Sweden; b Linköping University, Neurobiology, Sweden; c Region Jönköping County Hospital, Department of Clinical Physiology, Sweden; d Kalmar County Hospital, Department of Paediatrics, Sweden; e Örebro University Hospital, Department of Neurology and Rehabilitation, Division of Clinical Neurophysiology, Sweden; f Jönköping University, School of Health and Welfare, Sweden

**Keywords:** Sleep EEG, Melatonin, Sleep deprivation, Epilepsy, Children, Epileptic activity

## Abstract

**Objective:**

To evaluate possible age-specific differences in the diagnostic yield of sleep-deprived, and melatonin-induced pediatric sleep EEGs. The multicenter study was performed prospectively.

**Methods:**

Children referred to sleep EEG (n = 169), were stratified into three age groups (2–5; 6–11; 12–17 years) and randomized to sleep induction by melatonin or partial sleep deprivation. Epileptiform activity, sleep, adverse effects, and technical recording quality were compared between study arms and age categories.

**Results:**

Epileptiform activity occurred in 36% of the children, without significant differences between melatonin and sleep-deprived EEGs. In 6–11-year-old children, epileptiform abnormalities occurred in 49%, significantly higher than in the other age groups independent of the sleep-inducing method. Sleep, but not the sleep induction method, was significantly associated with the presence of epileptiform activity (OR 9.16). The occurrence of sleep was significantly higher (97%) after melatonin induction compared to sleep deprivation (86%) without age differences. No serious adverse effects were registered.

**Conclusions:**

There were no differences concerning the occurrence of epileptiform activity in EEG after melatonin induction or sleep deprivation in children of any age category. Melatonin increased the likelihood of sleep.

**Significance:**

Melatonin as a premedication for sleep EEG should be considered safe and efficient in terms of diagnostic yield.

## Highlights

• Epileptiform activity does not differ between melatonin-induced or sleep-deprived EEG in children.
• Epileptiform activity in EEG was associated with sleep but not the sleep-inducing method.
• Melatonin gives a higher probability of sleep compared to sleep deprivation and safe for EEG in children of different ages.

## 1 Introduction

Electroencephalography (EEG) is an important method in epilepsy diagnosis and is helpful in the classification of epilepsy syndromes (Koutroumanidis et al., 2017). EEG has high specificity for epilepsy (78–98 %), (Smith, 2005) but low sensitivity in children (18–56 %) (Wirrell, 2010). The diagnostic yield of EEG increases with different activation methods, including sleep (Kane et al., 2014, Marinig et al., 2000, Salinsky et al., 1987). EEG during sleep also minimizes movement artifacts, which is especially important in children (Flink et al., 2002).

Traditionally, the most common way to achieve sleep during EEG is through sleep deprivation, (i.e., keeping the patient awake for at least part of the night before the examination). However, the method is challenging to implement in children and usually implies that at least one caregiver will be sleep-deprived too. Another way to induce sleep is to use medication. Sedatives like chloral hydrate or benzodiazepines like midazolam are efficient but have adverse effects and can reduce epileptiform activity (Ashrafi et al., 2010, Fallah et al., 2014, Gumus et al., 2015).

Melatonin has been used to induce sleep in children during the last few decades (Alix et al., 2018, Eisermann et al., 2010, Wassmer et al., 2001a, Wassmer et al., 2001a, Wassmer et al., 2001b). Melatonin facilitates the transition from wakefulness to sleep but otherwise does not affect sleep (Zisapel, 2018). Previous studies showed that melatonin was well tolerated by children and that it induced sleep in 77–80 % of subjects (Alix et al., 2018, Eisermann et al., 2010, Wassmer et al 2001a). We have previously found that the efficacy of melatonin in inducing sleep is higher in younger children compared to older children (Gustafsson et al., 2015).

According to recommendations of the International League Against Epilepsy melatonin can be used for sleep EEG recordings in children up to 12 years of age, although the evidence is rather weak due to study limitations (small sample sizes and absence of reports concerning adverse effects) (Peltola et al., 2023).

Observational studies have shown no significant differences concerning the occurrence of epileptiform activity between sleep EEGs after melatonin induction vs. sleep deprivation in children (Alix et al., 2018, Gustafsson et al., 2015, Wassmer et al., 2001a). However, no randomized studies have analyzed age-dependent differences and possible adverse effects of melatonin. There are several reasons why age might matter: first, different epilepsy syndromes tend to debut at different ages and may also differ in their relative wake vs. sleep EEG sensitivities (Moore et al., 2021). Second, both the homeostatic sleep drive and the circadian sleep propensity may change during development (Campbell et al., 2024).

To assess whether melatonin can be safely used as an alternative to sleep deprivation in children, the present multicenter study was performed prospectively, aiming to evaluate possible age-specific differences in the occurrence of sleep, epileptiform activity and side effects.

### 1.1. Primary outcome

1.1

Is there a difference in the occurrence of epileptiform activity after sleep deprivation or melatonin intake in children who undergo sleep EEG due to suspected or diagnosed epilepsy?

### 1.2 Secondary outcomes

1. Are there differences in the occurrence of sleep, technical recording quality, and the occurrence of adverse effects during or after EEG after sleep deprivation and melatonin intake?
2. Are there any age-dependent differences concerning the occurrence of epileptiform activity, sleep, adverse effects, and technical recording quality of EEGs between EEG after sleep deprivation and melatonin intake in children aged between two and 17 years?

## 2 Methods

### 2.1 Trial design

These are data from a prospective, randomized controlled multicenter single-blind clinical trial. The study protocol was preregistered in Eudra CT (2016-000606-11). The trial included children two to 17 years old with their caregivers. The children were referred to sleep EEG due to suspicion of epilepsy or epilepsy follow-up. The children were divided into three age categories: 2–5 years, 6–11 years, and 12–17 years old. They were 1:1 block randomized to parallel groups: partial sleep deprivation (SD-EEG) or melatonin premedication (M- EEG) before the EEG. Block sizes varied randomly and were balanced in terms of age category and study center. The evaluator was blinded to the patients' clinical history and epilepsy diagnosis to ensure unbiased assessment.

### 2.2 Participants

**Fig. 1:**
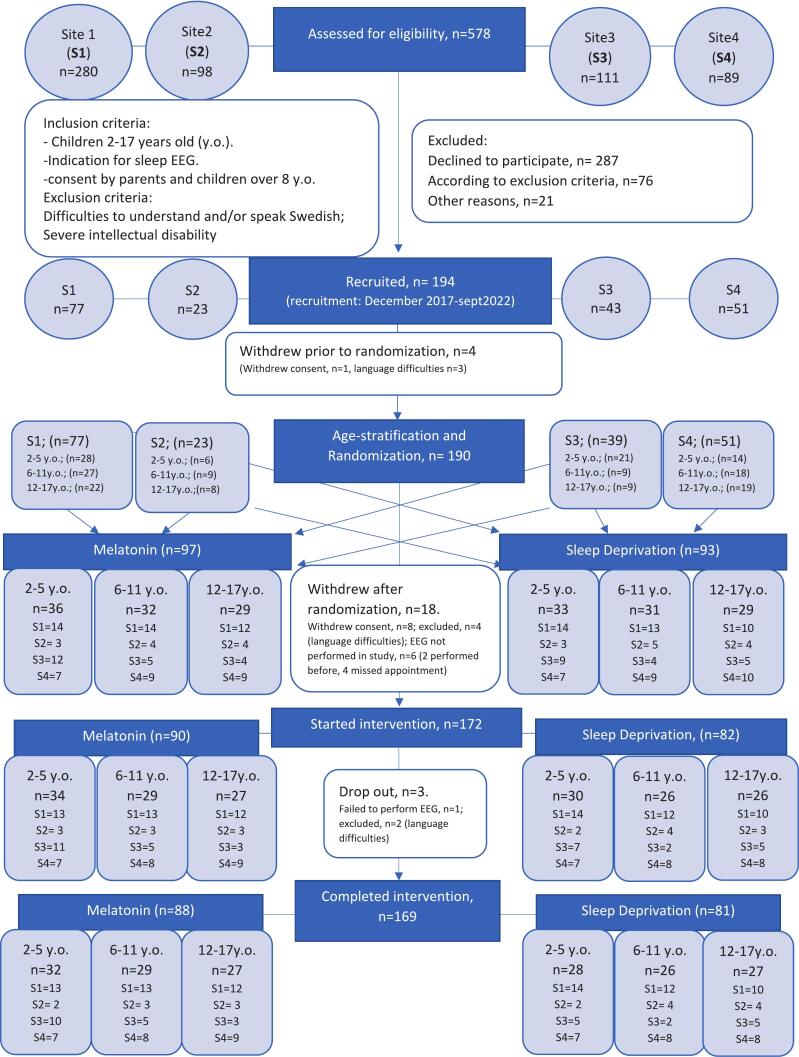
Flowchart recruitment to the study.

The clinical trial was started in December 2017 and ended in September 2022. Three centers were involved: the Neurophysiology Department at Linköping University Hospital (Site 1), the Physiology departments at Kalmar County Hospital (Site 2), and Jönköping County Hospital (Site 3). Due to low recruitment during the pandemic years one more center, the Neurophysiology Department in Örebro University Hospital (Site 4), was included from 2021 (Fig. 1).

All children two to 17 years old referred to sleep EEG were eligible to participate. Written consent from children older than eight years and from their parents was required for participation. All children got customized information about the study that could be read by the parent. Exclusion criteria were severe intellectual disability or severe autism and inability to understand and/or speak Swedish. In addition, children randomized for M-EEG were excluded if they had a known or suspected allergy to melatonin; liver or kidney failure; pregnancy or ongoing breastfeeding; as well as ongoing treatment with quinolones, fluvoxamine, 5- and 8-methoxypsoralen, cimetidine, or estrogens. To avoid melatonin premedication in pregnancy, all postmenarchal girls not using contraception provided a urine sample for a pregnancy test before the examination.

Enrollment took place either via a pediatrician/child neurologist in connection with the patients´ clinical visit, or via the EEG laboratory in those cases when physicians were not familiar with the study. After receiving the referral for sleep EEG, written information about the study was sent to potential study participants.

After receiving written consent from all participants age-stratification and randomization to either SD-EEG or M-EEG took place.

### 2.3 Interventions

Children randomized to SD-EEG were instructed to go to bed two hours later than their usual bedtime the night before the examination and to rise two hours earlier than their usual rising time on the morning of the examination. They were not allowed to sleep from the time they arose in the morning until the start of the examination. All analyses were performed on an intention-to-treat basis.

Children randomized to M-EEG were instructed to sleep as usual the night before. They received melatonin as an oral solution (1 mg/ml) mixed in a caffeine-free drink 15 min before the planned start of the examination. Children between the ages of two and four received 3 mg of melatonin, while children five years and older received 6 mg. Melatonin dosage was based on studies by Wassmer et al., 2001a and local clinical routine since 2009 (Gustafsson et al., 2015).

All children were instructed to avoid caffeinated drinks before the examination.

EEGs were performed according to clinical routines which were similar for all involved laboratories and followed the International Federation of Clinical Neurophysiology (IFCN) technical guidelines for EEG recording (Flink et al., 2002, Peltola et al., 2023). All EEGs, regardless of randomization method, were performed in the afternoons in a room with dim lighting and quiet surroundings. The children were asked to lie still, close their eyes, and try to fall asleep. EEG electrodes were placed on their head according to the 10–20 international system.

All EEG assessments were performed by one physician (first author), a specialist in clinical neurophysiology, who was blinded regarding the sleep induction method. Blinding for children or caregivers was impossible given that only the SD-EEG group was supposed to be sleep-deprived.

The EEG assessment included background activity, epileptiform activity, non-epileptiform abnormalities (i.e., focal and/or general slowing, asymmetry, and focal changes). Assessment of sleep was evaluated in accordance with American Academy of Sleep Medicine guidelines (Iber et al., 2007).

Technical recording quality was assessed based on the presence of artifacts in the EEG during wakefulness and sleep. Poor technical quality was defined as artifacts affecting the interpretation that was present for 50 % of more of the recording duration.

In connection with the EEG visit, children and caregivers received a questionnaire asking about unwanted events up to 24 h post-examination.

All incidents that occurred during the EEG and up to 24 h post-examination, regardless of whether they were related to melatonin or not, were recorded and documented on a special adverse events (AE) report that was evaluated and signed by a physician.

### 2.4 Statistical methods

The sample size was based on a power calculation that in turn was based on a non-inferiority analysis of a dichotomous outcome variable (the presence of epileptiform activity) and prevalence data for epileptiform activity in the corresponding patient population (same age, same indications, same melatonin doses and same participating clinics) in a previously conducted retrospective study (Gustafsson et al., 2015). The study was originally planned to recruit 300 children. Due to the low recruitment rate, the study ended prematurely after five years with 169 recruited children.

All statistics were performed in R (The R Foundation for Statistical Computing, Vienna, Austria). Basic descriptive statistics are presented as absolute and relative frequencies and median and quartiles. Univariate analyses of between-group differences were performed with a chi-square test and Fisher’s exact test for categorical data. For continuous data, a T-test and Wilcoxon’s rank sum test were used, depending on probability distributions. To compare continuous variables across study sites or between different age categories, a Kruskal-Wallis H test was used.

To explore factors affecting the diagnostic yield of EEG in children of different ages (i.e., the presence of epileptiform activity, technical recording quality and sleep), logistic regression was employed. The choice of independent variables was made based on theoretical assumptions and on the results from univariate analyses.

The study was approved by the Swedish Ethical Review Authority (Dnr 2015/164-31, Dnr 2020-01563).

## 3 Results

### 3.1 Sample description

190 children were included and randomized to the study, (Fig. 1).

169 children completed the study, 90 boys (53 %) and 79 girls. They were randomized into M-EEG (n = 88 (52 %)), and SD-EEG (n = 81 (48 %)) groups. The median age was eight years (Q1-Q3 5–12 years). Twenty-nine children (17 %), without significant differences between study arms or age groups, had previously known neurological, neurobehavioral, or intellectual comorbidity at the time of the EEG. Twenty-eight children (17 %) (14 in each study arm) had anti-seizure medication (ASM) at the time of the EEG. Sixty-four (48 %) children had undergone EEG prior to the study. Of those recordings, 42 (66 %) were normal. There were no significant differences in the distribution of first/ non-first EEG recordings between intervention arms or between age categories. The missingness of clinical data was low in the whole sample (6 %) without significant differences between M-EEG and SD-EEG or in different age groups.

Age-specific demographic data are presented in Table 1.

**Table 1:** Demographic and clinical background data of study participants, according to age and intervention.

	2–5 years old			6–11 years old			12–17 years old		
EEG method	M-EEG	SD-EEG	**Total**	M-EEG	SD-EEG	**Total**	M-EEG	SD-EEG	**Total**
Number, n,	32	28	**60**	29	26	**55**	27	27	**54**
Boys, n	19	15	**34**	15	13	**28**	13	15	**28**
Median age Q1-Q3	3 2–5	4 3–5	**4** **3–5**	9 7–10	8 7–9	**9** **7–10**	14 13–15	15 13–16	**14** **13**–**15**
Seizure type Bilateral, n Focal, n Absence, n	10 4 *a 15*b	9 11*b 6	**19** **15** **21**	7 15 5	5 13 8	**12** 28*c **13**	8 11 6	10 10 5	**18** **21** **11**
ASM, n	2	3	**5**	7	5	**12**	5	6	**11**

Footnote: *a– Significant (p < 0.05) difference between different age-groups in the same EEG-method (M-EEG or SD-EEG). *b- Significant (p < 0.05) difference between EEG methods in an age group (M-EEG vs SD-EEG). *c – Significant (p < 0.05) difference between age-groups in total, regardless of sleep induction method (M-EEG + SD-EEG).

All EEGs started between 12:47 to 13:46, median time 13:10 (Q1-Q3, 13:02-13:18). The EEG recordings had a median duration of 47 min (Q1-Q3, 40-52). No differences in the parameters above were registered between study arms or age groups. Forty children (24 %), almost all from site 4, underwent provocation (photic stimulation, hyperventilation) during the EEG recordings which did not affect outcomes.

Only one sleep-deprived child slept before the EEG, according to parental reports.

### 3.2 Primary outcome: Epileptiform activity

Epileptiform activity was identified in 36 % (n = 60) of children in the whole sample. The proportion of epileptiform discharges was slightly higher at 38 % (n = 34, n.s.) in M-EEGs compared with SD-EEGs at 32 % (n = 26). The occurrence of epileptiform discharges was significantly higher, at 49 % (p < 0.05) in 6–11 years old children, compared to other ages (22 % in children 2–5 years old, 37 % in children 12–17 years old,) regardless of the sleep induction method (Table 2). Focal epileptiform activity was significantly more common (52 %, p < 0.05) compared to generalized (15 %) or multifocal (33 %) (Table 2).

**Table 2:** Age- specific EEG results.

Variables	2–5 years old			6–11 years old			12–17 years old		
EEG method	M-EEG	SD-EEG	**Total**	M-EEG	SD-EEG	**Total**	M-EEG	SD-EEG	**Total**
Number, n = 169	32	28	**60**	29	26	**55**	27	27	**54**
Normal EEG, n = 100	25	22	**47**	13	11	**24**	12	17	**29**
EA: Total, n = 60	7	6	**13**	16*a	11	27*b	11	9	**20**
EA generalized, n = 9	3	0	**3**	1	2	**3**	1	2	**3**
EA focal, n = 31	2*a	5	**7**	9	5	**14**	7	3	**10**
EA multifocal, n = 20	2	1	**3**	6	4	**10**	3	4	**7**
EA during: Sleep only, n = 31 Wake only, n = 3 Sleep and wake, n = 26	4 0 3	3 0 3	**7** **0** **6**	7 0 9*a	3 3 5	**10** **3** 14*b	8 0 3	6 0 3	**14** **0** **6**
Artefacts awake, n = 25	11*a	7	18*b	4	2	**6**	1	0	**1**
Artefacts sleep, n = 8	5	3	**8**	0	0	**0**	0	0	**0**
Occurrence of sleep, n = 155	30	24	**54**	28	22	**50**	27	24	**51**
Children achieved NREM sleep stage: N1: n = 155 N2: n = 153 N3: n = 117	30 30 27	24 24 23	**54** **54** **50**	28 28 20	22 21 20	**50** **49** **40**	27 27 15*a	24 23 12*a	**51** **50** 27*b

Footnote: “EA”- epileptiform activity. *a– Significant difference (p < 0.05) between different age-groups in the same EEG-method (M-EEG or SD-EEG); *b-Significant difference (p < 0.05) between age-groups in total, regardless of sleep induction method (M-EEG + SD-EEG).

**Fig. 2:**
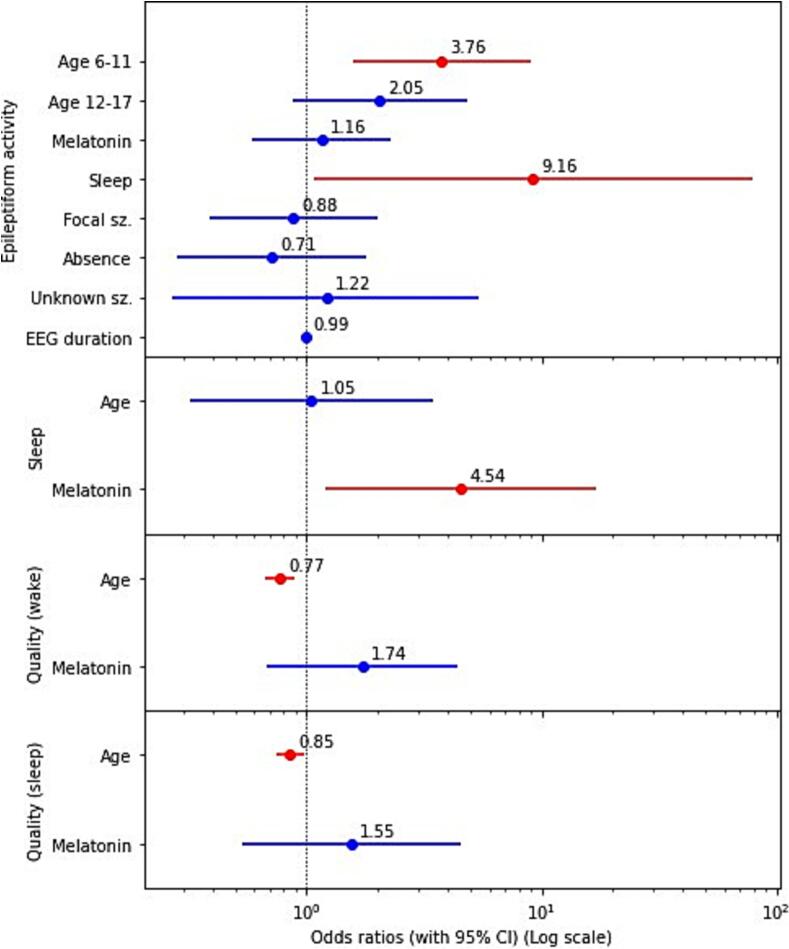
Predictors of epileptiform activity, sleep and EEGs recording quality. Footnote: “sz”- seizure. Red color indicates significant difference (p < 0.05).

When epileptiform activity occurred, it was recorded exclusively during sleep in 52 % (n = 31) of the cases and in 5 % (n = 3) during wakefulness only (Table2). Twenty-nine children (17 %) had background abnormalities in EEG without differences between study arms or age groups. Seizures were rare (n = 3, 2 %). They occurred during wakefulness (in one child before sleep, in another one-after sleep, and one child had absence during hyperventilation after sleep). All children were sleep deprived. Logistic regression showed that epileptiform activity was significantly more likely in children 6–11 years old (OR 3.76, 95 % CI 1.58–8.94 compared to 2–5-year-old children). The occurrence of sleep, but not the sleep induction method, was significantly associated with the presence of epileptiform activity (OR 9.16, 95 % CI 1.07–78.5) (Fig. 2).

### 3.3 Secondary outcomes

#### 3.3.1 Technical quality

The number of EEGs of poor technical quality was significantly higher (p < 0.05) during wakefulness (15 %, n = 25) compared to sleep (5 %, n = 8) in all children, and in 2–5-year-old children compared to other age categories (Table 2). Artifacts during sleep were seen only in 2–5-year-old children. There was no significant difference concerning artifacts in M-EEGs (n = 16, 18 %) vs. SD-EEGs (n = 9, 11 %).

#### 3.3.2 Sleep

Sleep occurred in 92 % (n = 155) of all children, with a significantly higher (p < 0.05) proportion in M-EEGs (97 %, n = 85) compared to SD-EEGs (86 %, n = 70). No significant differences in sleep occurred between the different age categories (Table 2). There were no significant differences in sleep onset latency (median 13 min; Q1-Q3, 5–16) and sleep duration (median 32 min, Q1-Q3, 25–36) between study arms or age categories. Significantly less (p < 0.05) older children (13–17 years old) reached N3 NREM sleep (n = 27, 50 %) compared to younger: 2–5 years old (n = 50, 83 %); and 6–11 years old (n = 40, 73 %) without significant difference between study arms (Table 2). Logistic regression indicated that children who underwent M-EEG were significantly more likely to fall asleep than children who underwent SD-EEG (OR 4.54, 95 % CI 1.21–16.96) (Fig. 2).

#### 3.3.3 Adverse events (AE)

AE are presented in Table 3.

**Table 3:** Adverse events (AE) in different age-groups.

Variables	2–5 years old		6–11 years old		12–17 years old	
EEG method	M	SD	M	SD	M	SD
Number of children with AE, n = 45	9*	1	10	8	8	9
AE in total, n = 61#: Headache n = 22 Stomach-ache, n = 10 Vertigo, n = 9 Other, n = 20	10* 1 1 2 6	1 0 0 0 1	14 7 3 1 3	11 6 1 1 3	14 4 3 2 5	11 4 2 3 2

Footnote: *– Significant difference (p < 0.05) between different age-groups in the same EEG-method (M-EEG or SD-EEG).

#- some participants reported more than one symptom. Other reported AE were: migrane-2; itching-2; fever-2; nausea- 2; sleep disturbances-3; problem in school-1; tics-1; cold −1; pain in eyes-1; electrode marks-1; unspecified- 4.

No serious AE were registered. All reported AE were mild and resolved within a few days with no consequences. We did not receive responses from nine (5 %) participants.

Seventy-three (43 %) children or their caregivers reported symptoms 24 h after the EEG examination. Tiredness was the most frequently reported symptom (n = 51, 30 %) and in 28 (17 %) cases it was reported as a single event without significant differences between study arms or age groups. Since tiredness was an expected consequence of the procedure, the symptom was not reported as an adverse event. Children who received melatonin premedication reported adverse events slightly more often (31 %, n = 27, n.s.), than sleep-deprived children (22 %, n = 18).

## 4 Discussion

This study compared two methods of sleep induction for sleep EEG in children, demonstrating no significant differences concerning the occurrence of epileptiform activity between melatonin-induced sleep and partial sleep deprivation, in EEG recordings for the study population as a whole.

Possible age differences were explored, and we found that children 6–11 years old had a significantly higher incidence (49 %) of epileptiform discharges compared to the other age categories regardless of the sleep induction method. In this age category, focal seizures were registered more often (51 %) compared to children of other ages. We have reported similar results in a recently published large retrospective study (Gustafsson et al., 2025). A likely explanation is that self-limited epilepsies usually debut in this age group (Wirrell, 2010).

Focal epileptiform activity was recorded in a higher proportion of children than other types of epileptiform discharges. Previous studies have shown that focal epileptiform discharges are better revealed during sleep than during wakefulness (Moore et al., 2021, Wirrell, 2010). Children with focal seizures and clinical symptoms of self-limited epilepsy can therefore benefit from undergoing sleep EEG. Regarding the prevalence of generalized epileptiform activity, it is important to note that only children who were planned for sleep EEG were approached for possible study inclusion. Thus, in cases where the referring physician wanted a wake EEG (e.g., CAE suspicion), children were not included in this study. This can affect the relative prevalence of focal vs. generalized epileptiform activity in this sample.

The proportion of epileptiform discharges in the whole sample was 36 %, which is in accordance with our previously reported results and with other studies (Gustafsson et al., 2015, Wirrell, 2010). The occurrence of epileptiform abnormalities usually varies in studies, and one of the reasons may be differences in study design (Giorgi et al., 2013). In the present study we included a heterogenous sample of children, both with new-onset seizures and with established epilepsy diagnoses. This is one of the study limitations and could potentially bias the results.

Sleep was obtained significantly more often with melatonin premedication compared to sleep deprivation (97 % vs 86 %), with no differences between age groups. Other studies have also shown a good sleep-inducing effect of melatonin in children (Alix et al., 2018, Wassmer et al., 2001a). In a previous retrospective study (Gustafsson et al., 2015) we found better sleep-inducing effects of melatonin in young compared to older children. We expected to find similar results, as endogenous melatonin production rises in early infants and remains stable until puberty when production declines (Cavallo and Ritschel, 1996). Surprisingly, melatonin showed as good sleep-inducing effect in the older age categories as in the youngest children in the present study. This could be due to differences in study design: in the retrospective study, we did not exclude children with severe neurological and neurobehavioral comorbidity, which we did in this study. As they may have more difficulties to fall asleep during an EEG, (Eisermann et al., 2010) this may have led to a seemingly lower efficiency of melatonin in the retrospective study. This is also one of the limitations of the present study. Children with neurological and intellectual deficit are difficult to investigate but EEG might be crucial for epilepsy diagnosis and further treatment in these patients. Using more potent medication or combining with sleep deprivation might be required to achieve successful sleep EEGs. Güzin et al., 2025 showed good sleep-inducing effect of chloral hydrate compared to hydroxyzine and melatonin in paediatric sleep deprived EEGs. Unfortunately, many drugs have side effects, can interfere EEG activity or need monitoring during and after EEG (e.g. dexmedetomidine) (Chen et al., 2020, Güzin et al., 2025). Data about combining sleep deprivation with melatonin is, however, rather controversial. Alix et al. (2018) reported sleep occurrence in 90 % of patients after combining sleep deprivation and melatonin which was more advantageous than using melatonin alone. In contrast, Sander et al., 2012 reported no additional sleep-inducing effects of melatonin in sleep-deprived children.

Regardless of the sleep induction method, sleep was recorded in 92 % of the investigated children, which is slightly higher than previously reported (77–80 %) (Alix et al. 2018, Eisermann et al., 2010). One of the reasons why the proportion of obtained sleep was higher in the present study could be that EEGs were performed in the afternoon while traditionally sleep EEGs are performed during morning hours. Afternoon recordings were performed in the present study since we wished to perform EEGs at the same time of day, regardless of the sleep-inducing method. We assumed that melatonin would have a better effect during afternoons due to increasing endogenous melatonin levels (Waldhauser et al., 1988). The results of the present study with a high proportion of obtained sleep indicate that it may be at least as advantageous to perform sleep EEGs in the afternoon as during the morning hours.

One of the centers (site 4, Fig. 1) had a higher recruitment rate (53 %) compared to the other sites (23 %-39 %), which might have been because melatonin was introduced as a new method for sleep induction at this site, while the other sites were already using melatonin in their routines for sleep EEG. We believe that preferring melatonin premedication instead of sleep deprivation was considered as more convenient for parents and their children and might explain the higher recruitment at this site.

There were no serious adverse events after melatonin premedication. Only mild symptoms were reported. Children two to five years old more often reported AE after melatonin premedication compared with those who were sleep deprived. No differences in adverse events in other age categories were seen. However, mild AE after melatonin intake have been reported previously without comparisons between ages or sleep deprivation (Sander et al., 2012, Wassmer et al., 2001b).

The incidences of muscle and other types of artifacts were relatively low. There were significantly more artifacts recorded in young children compared to older ones. This could be a practical reason to avoid wakefulness in young children. We and other authors have previously reported similar results, i.e., up to 10 % of unsuccessful EEG recordings because of artifacts (Gustafsson et al., 2015, Sander et al., 2012).

## 5 Conclusion

This randomized controlled multicenter single-blind clinical trial showed no differences in EEGs when sleep was induced by melatonin or partial sleep deprivation as regards the occurrence of epileptiform activity, quality of recordings, or adverse events, but sleep was obtained more often after melatonin induction. Epileptiform activity in EEG was associated with sleep but not with the sleep-inducing method. There were no differences between ages except for children six to 11 years old, who had a significantly higher incidence of epileptiform discharges compared to the other age categories.

The study was approved by the Swedish Ethical Review Authority (Dnr 2015/164-31, Dnr 2020-01563).

The study was conducted according to the clinical trial protocol, LVFS 2011:19, ICH-GCP and the latest version of the Declaration of Helsinki, i.e. the version from 2013. Registration number EudraCT 2016-000606-11.

The study was conceptualized by MU, ES, MV and AB. The study was conducted by MV, CL, JT, KD and GG. The main author is GG. Analyses of data were performed by MU, MV and GG. The initial draft of the manuscript was written by GG and MU. All authors contributed to the revision and the final version of the manuscript.

## Funding

Research Council of Southeastern Sweden (FORSS) (grant number: FORSS-344791; FORSS-387401; FORSS-754131; FORSS-932075).

Region Östergötland, ALF (grant number: RÖ-355 441; RÖ-445 321; RÖ-699401; RÖ-606661; RÖ-607171).
